# The Placenta as a Target Organ for Poly- and Perfluoroalkyl Substances (PFASs): Molecular Mechanisms of Toxicity

**DOI:** 10.3390/ijms27042036

**Published:** 2026-02-22

**Authors:** Paola Inés Ingaramo, Maria Laura Zenclussen

**Affiliations:** 1 Instituto de Salud y Ambiente del Litoral (ISAL), Universidad Nacional del Litoral (UNL)–Consejo Nacional de Investigaciones Científicas y Técnicas (CONICET), Santa Fe 3000, Argentina; pingaramo@fbcb.unl.edu.ar; 2 Cátedra de Fisiología Humana, Facultad de Bioquímica y Ciencias Biológicas (FBCB), Universidad Nacional del Litoral (UNL), Santa Fe 3000, Argentina; 3 Cátedra de Fisiología, Facultad de Ciencias Médicas (FCM), Universidad Nacional del Litoral (UNL), Santa Fe 3000, Argentina

**Keywords:** PFAS, placenta, oxidative stress, NAMs

## Abstract

Exposure to poly- and perfluoroalkyl substances (PFASs) has been a cause for concern for decades due to evidence linking exposure to these substances with adverse health effects. Its widespread use in industrial and consumer products combined with their persistence in the environment poses a toxicological and regulatory challenge. Due to its ubiquity, resistance to degradation, and accumulation in biological systems, humans are exposed to a mixture of multiple PFASs, which complicates the analysis of exposure effects. As PFASs pose a risk to human health, it is crucial to study their impact during vulnerable periods, such as pregnancy. In this regard, understanding the impact of PFASs on the placenta is essential, as they can affect both pregnancy and the well-being of the developing fetus. This article reviews the current evidence linking PFAS exposure with altered placental function, focusing on the affected molecular pathways. Furthermore, we examine current methodologies for analyzing the effects of exposure to complex mixtures and explore how these approaches could be employed to evaluate the potential impact of such mixtures on placental function in the context of real-life exposure to these chemicals.

## 1. Introduction

Poly- and perfluoroalkyl substances (PFASs) are a family of highly fluorinated aliphatic compounds, containing at least one fully fluorinated carbon moiety such as –CF_3_ or –CF_2_– in the molecular structure [1]. Perfluoroalkyl substances are those with fully fluorinated carbon chains, whereas polyfluoroalkyl substances have only partial substitution of hydrogen atoms by fluorine [2]. The carbon–fluorine (C-F) bond endows these compounds with remarkable stability and their most valuable industrial properties: water and oil repellency [3,4]. Nevertheless, these exceptional properties are the reason for their remarkable resilience to environmental, biological and thermal degradation [3]. They are used in everyday products such as food packaging, cookware, textile stains and furniture [2]. In addition, they are used in industrial processes such as the manufacture of coatings and fluoropolymers, and in aqueous film-forming foams, which are frequently used for firefighting [3].

Their widespread use and extreme persistence have led to environmental contamination, guaranteeing their entry into the food chain and water supplies. This has resulted in a measurable body burden worldwide. The presence of these compounds in humans has been associated with adverse health effects, with most of the studied effects being linked to perfluorooctane sulfonate (PFOS) and perfluorooctanoic acid (PFOA) [5]. These are both polyfluorinated compounds (PFCs), which are considered long-chain PFASs. These substances are part of a group known as legacy PFASs and include widely used compounds such as perfluorooctanoic acid (PFOA) and perfluorooctane sulfonate (PFOS). These compounds are now regulated due to their persistence in the environment, their ability to bioaccumulate, and their adverse health effects [6]. These effects include metabolic disturbances, immune system disturbances and endocrine disruption, among others (reviewed in [7]). Consequently, various programs have been implemented worldwide to reduce the use of the most prevalent environmental PFASs, and the production of long-chain PFASs has declined. This has led to a decrease in exposure to PFOS and PFOA in certain regions due to regulatory interventions. Nowadays, the manufacturing industry has moved on to shorter-chain-length PFASs, which are thought to be less harmful than their predecessors. These new alternatives, nowadays referred to as “emerging PFASs”, are short-chain PFASs that have been adopted by the chemical industry as alternatives to long-chain PFASs [6,8]. However, studies analyzing some of these new PFASs suggest that they are just as hazardous [9].

As some of the effects of PFASs on human health are related to endocrine disruption, it is crucial to study their impact during vulnerable periods, when the negative effects of endocrine disruptors are most pronounced. One such period is pregnancy, as the effects on developing children may be evident in adulthood. While most studies focus on the effects of single exposures, there is still a need to understand the effects of exposure to combinations of different PFASs, as occurs in real life. Furthermore, given the vulnerability of fetuses and newborns, it is crucial to understand the potential negative effects of exposure to multiple PFASs on pregnant people and their developing children. As placental health is crucial for fetal development, special attention should focus on the effects of PFASs on the placenta, as it is a direct target of PFAS exposure [10].

Here, we will summarize the current evidence of the impact of exposure to PFASs (either alone or in mixtures) on placental function, emphasizing the affected molecular pathways and their effect on pregnancy outcomes. Furthermore, we will discuss the methodologies currently used to analyze complex mixtures and how these could be employed in the future to study the effects of exposure considering real-life scenarios. This will help in the development of new regulatory measures aimed at avoiding concomitant exposure to PFASs.

## 2. Prenatal and Preconception PFAS Exposure and Pregnancy Outcomes

Zhang et al. [11], reported dose-dependent associations between first-trimester maternal serum concentrations of PFOA, PFOS, perfluorohexane sulfonic acid (PFHxS), and perfluorononanoic acid (PFNA) (median of 5.8 ng/mL, 25.2 ng/mL, 2.4 ng/mL and 0.7 ng/mL, respectively) and reduced birth weight, even at relatively low exposure levels. Notably, these associations were confined to mothers with low dietary or plasma folate concentrations, suggesting that adequate folate status may partially mitigate PFAS-related fetal growth impairment. Consistent with the overall exposure evidence, legacy and emerging PFASs have been consistently detected in maternal serum and plasma, fetal serum, umbilical cord blood, and placental tissue, indicating widespread maternal–fetal exposure [12,13,14,15,16]. Population-based monitoring studies have demonstrated the ubiquitous presence of PFASs in human serum, with detection frequencies exceeding 90% in the general population [5], characterized by high detection rates of legacy PFASs and comparatively lower detection of emerging PFASs [5,12]. This pattern is confirmed across multiple biological matrices in pregnancy cohorts and comprehensive reviews [17] and supported by evidence linking prenatal PFAS exposure with pregnancy outcomes [13,14,15,16,17,18]. EFSA has established a very low tolerable weekly intake of PFASs (4.4 ng/kg body weight per week), based on effects observed in children. This corresponds to an estimated maternal serum concentration of 6.9 ng/mL. According to Jaus et al. [12], 41% of women of reproductive age and 74% of the entire studied population exceed this level. In other words, exposure levels sometimes exceed the limits established by regulatory agencies, depending on population characteristics, age, and occupational status. In placental samples (decidua and villi samples) of pregnant people at term, recruited from hospitals in Italy, the median total PFAS values ranged from 0.16 to 2.39 ng/g [8]. PFASs were detected in 95.7% of samples, with PFOS being the most frequently detected compound (88%), followed by PFHxS (83%), PFOA (83%), perfluorobutane sulfonic acid (PFBS) (54%), and perfluorohexanoic acid (PFHxA) (54%) [14]. This data further supports the placenta as a key site of PFAS accumulation and a critical interface for maternal–fetal transfer. Indeed, placental transfer studies have demonstrated selective transplacental passage of classical PFASs, dependent on carbon chain length and functional group chemistry, with consistent detection in fetal serum and umbilical cord blood [15,16]. Likewise, Jingwen Jia et al. [13] demonstrated the presence of PFASs in the umbilical serum, indicating that chemicals could cross the placental barrier. In this work, the detection rates of emerging PFASs such as PFBS, perfluorobutanoic acid (PFBA), perfluoroheptanoic acid (PFHpA), perfluorodecanoic acid (PFDA), perfluorodecane sulfonic acid (PFDS), and perfluorohexane sulfonamide (FHxSA) were low (2%, 5%, 21%, 1%, 12%, and 6%), while legacy PFASs such as PFNA, PFOS, perfluoroundecanoic acid (PFUdA), pentafluoropropionic acid (PFPrA), and PFHxS were detected at >70% [13]. The concentration ranges of short-chain and long-chain PFASs were 0.000–7.714 ng/mL and 0.117–3.990 ng/mL with 1.092 ng/mL being the highest median concentration of PFOS in umbilical serum and being negatively associated with neonatal birth weight [13]. Although most of the reported adverse effects have been primarily associated with legacy PFASs, evidence regarding emerging PFASs remains limited. While these newer compounds often exhibit comparatively shorter biological half-lives, this does not necessarily imply lower toxicity, underscoring the need for further epidemiological and mechanistic studies to adequately characterize their health risks. Across diverse population-based cohorts from North America and Europe, PFOSs, PFNA, PFHxS, and PFOA have been detected in more than 50% of pregnant women, with total PFAS concentrations frequently exceeding 2 ng/mL in serum or plasma [19]. These exposures have been significantly associated with biomarkers of oxidative stress and may also contribute to long-term metabolic alterations, including increased risk of type 2 diabetes later in life [19,20]. In a Wisconsin case–control study, higher maternal serum concentrations of PFOS and perfluorohexane sulfonate (PFHPS) were associated with a markedly increased risk of preeclampsia, with interquartile range increases corresponding to 7.18- and 5.40-fold higher odds of disease, respectively [21]. These associations were accompanied by elevated circulating antiangiogenic markers, including soluble fms-like tyrosine kinase-1 (sFLT-1) and the sFLT-1/placental growth factor ratio. Mechanistic support for these epidemiological observations is provided by in vitro studies demonstrating that PFOS directly impairs endothelial angiogenic capacity through suppression of endothelial growth factor receptor 2 signaling, thereby linking maternal PFAS exposure to placental vascular dysfunction and hypertensive pregnancy disorders [21]. Beyond fetal growth outcomes, prenatal PFAS exposure at cumulative serum concentrations typically ranging from 2 to 20 ng/mL has been associated with broad alterations in maternal immune and metabolic pathways [18,22,23,24]. Several studies have reported inverse associations between PFAS exposure and circulating inflammatory cytokines, including Interleukin-10 (IL-10), Interleukin-6 (IL-6), and Tumor Necrosis Factor alpha (TNF-α), suggesting immune modulation rather than overt immune activation during pregnancy [22]. Dysregulation of these maternal inflammatory mediators has been linked to major pregnancy complications, including preterm birth, preeclampsia, and intrauterine growth restriction [22]. In parallel, PFAS exposure has been consistently linked to disruptions in lipid metabolism during early gestation [18,23]. Notably, increases in PFAS mixtures at concentrations ranging from 0.01 to 100 ng/mL during early-to-mid pregnancy have been associated with approximately 30% lower insulin and Homeostasis Model Assessment of Insulin Resistance (HOMA-IR) levels, alongside 7–10% higher high-density lipoprotein cholesterol concentrations [24]. These patterns reflect complex metabolic adaptations with uncertain long-term implications rather than clearly protective effects such as negative associations of certain PFASs with insulin and leptin that may reflect reduced fetal energy availability [24]. In women undergoing in vitro fertilization–embryo transfer, novel machine learning frameworks that integrated PFASs and metal(loid) mixture exposures, as measured in hair, serum, and follicular fluid, predicted early pregnancy loss with high accuracy. These frameworks also identified key chemical drivers and disrupted biological pathways that are implicated in reproductive failure [25]. While these findings highlight the utility of integrative exposure modeling, external validation and causal inference remain necessary [25]. More recently, emerging PFASs, including perfluoroalkyl ether carboxylic acids (PFECA), polyfluoroalkyl ether sulfonic acid (PFESA), fluorotelomer sulfonic acid (FTS), hexafluoropropylene oxide dimer acid (HFPO-DA) and perfluoroalkyl phosphonic acid (PFPAs) have been identified in maternal and fetoplacental matrices, primarily through targeted and suspect screening approaches [6,26,27]. Chlorinated polyfluoroalkyl ether sulfonic acids, such as F-53B (CI-PFESA), have been detected in paired maternal serum, umbilical cord blood, and placental samples, providing direct evidence of transplacental transfer and placental bioaccumulation [26,28]. Although it is complex to reach a definitive verdict on PFAS toxicity at specific concentrations, given the influence of multiple factors, it is evident that the bioaccumulation of these compounds exerts toxic effects at multiple biological levels, underscoring the need to establish maximum safety guidelines during particularly sensitive periods such as pregnancy. Moreover, studies focusing on specific placental compartments and epigenetic endpoints have reported the presence of both legacy and emerging PFASs and linked them to histopathological alterations and perturbations in DNA methylation, reinforcing the placenta as a key toxicological target of emerging PFASs [14,27,29]. Together, these findings underscore the importance of accurately characterizing maternal and placental PFAS burdens to elucidate mechanisms of maternal–fetal transfer and susceptibility during critical windows of development. Table 1 summarizes selected legacy and emerging PFASs detected in maternal, placental or umbilical cord samples. Although PFOS and PFOA remain the most prevalent compounds, the growing diversity of alternative PFASs identified at the fetal–maternal interface highlights the increasing complexity of real-world exposure scenarios and poses significant challenges for risk assessment and regulatory frameworks.

## 3. Placenta as a Target—Mechanisms of Action

### 3.1. Cellular and Functional Alterations in In Vitro Trophoblast Models

Accumulating evidence from in vitro toxicological screens identifies the placenta as a key target of PFAS-mediated toxicity. Using a high-throughput approach in JEG-3 trophoblasts was demonstrated that exposure to a diverse panel of 42 PFASs produces measurable impairments in viability, proliferation, mitochondrial membrane potential, and migration [32]. While several PFASs assessed in this screening displayed well-defined concentration–response relationships consistent with classical toxicological models (e.g., PFOS, PFOA, PFNA), other compounds failed to show a clear dose-dependent pattern (e.g., GenX, perfluoropentanoic acid, perfluorohexanoic acid [32]. These functional disturbances are accompanied by dysregulation of oxidative-stress-related genes such as glutathione peroxidase 1 (GPX1), G protein-coupled Estrogen Receptor 1 (GPER1), Superoxide Dismutase 1 (SOD1) and transporters involved in xenobiotic handling as ATP-Binding Cassette Subfamily G Member 2 (ABCG2) [32]. This indicates that PFASs can alter trophoblast physiology even at non-cytotoxic concentrations [32]. Three-dimensional spheroid models further underscore the complexity of PFAS actions. In JEG-3 spheroids, high concentrations (300 μM) produce profound structural damage and fully suppress invasive behavior, whereas lower concentrations (0.1–100 μM) paradoxically enhance invasion [33]. Conversely, HTR-8/SVneo spheroids exhibit a more uniform sensitivity, with consistent inhibition of invasion at low–medium doses and minimal effects at the highest concentration tested [33]. Although some studies employed supraphysiological concentrations [33], concordant effects observed at submicromolar or nanomolar levels across multiple models support the biological relevance of these pathways [32,33,34,35]. Together, these findings suggest that even modest PFAS exposure can dysregulate trophoblast function across multiple biological axes. Beyond effects on viability and invasion, PFAS exposure also compromises trophoblast endocrine function, as PFAS mixtures reduce beta subunit of human chorionic gonadotropin (β-hCG) secretion and downregulate expression of its coding gene Chorionic Gonadotropin Beta Subunit 7 (CGB7) at concentrations as low as 0.01 μM [33]. Consistent with this endocrine reprogramming, inhibition of cAMP response-element-binding protein (CREB)-dependent transcription and suppression of peroxisome-proliferator-activated receptor gamma (PPARG) link altered hormonal signaling to impaired trophoblast differentiation and metabolic function [34,35].

### 3.2. Molecular and Transcriptomic Dysregulation of Trophoblast Differentiation and Placentation

Building on these functional observations, transcriptomic profiling provides convergent evidence that PFASs disrupt coordinated molecular programs that are essential for placental development. In JEG-3 spheroids, PFAS exposure downregulates key regulators of apoptotic balance such as BCL2-associated agonist of cell death (BAD), BCL2 antagonist/killer (BAK), B-cell lymphoma 2 (BCL2), and galectin-3, together with genes critical for trophoblast proliferation and placentation, including epidermal growth factor receptor, notch receptor 3, and placental growth factor [33]. In parallel, studies using HTR-8/SVneo models show increased expression of Caspase-3 (CASP3) and Insulin-Like Growth Factor 2 alongside reduced galectin-3 and macrophage migration inhibitory factor (MIF), reflecting altered apoptotic control and disruption of growth- and immune-modulatory pathways [33]. These patterns are consistent with earlier observations by Bangma et al. [36], who reported PFAS-induced modulation of transcriptional networks involved in syncytialization, nutrient transport, cell growth, and apoptosis, characterized by upregulation of proapoptotic genes (e.g., BAD, BAX) and downregulation of antiapoptotic BCL2, indicating a shift toward a more apoptosis-prone trophoblast state. Recent advances using human trophoblast organoid systems further refine these mechanistic insights by capturing lineage-specific effects of PFASs on placentation. Exposure to PFOA and PFOS alters trophoblast cell fate decisions, increasing villous cytotrophoblast proportions while reducing extravillous trophoblast and syncytiotrophoblast populations, with PFOA exerting effects at lower concentrations than PFOSs [34]. These changes are linked to inhibition of cAMP response element-binding protein (CREB)-dependent transcription, a pathway critical for trophoblast differentiation [34]. Functionally, disrupted lineage specification is accompanied by reduced hormone secretion and impaired invasive capacity, linking PFAS-induced transcriptional dysregulation to placental dysfunction across multiple functional domains, with potential consequences for adverse birth outcomes [33]. Importantly, these transcriptional perturbations also affect metabolic programming, as PFBS suppresses placental lipid metabolism by downregulating PPARG and key components of the carnitine shuttle as carnitine palmitoyltransferase 1A (CPT1A) and solute carrier family 25 member 20 (SLC25A20), thereby limiting mitochondrial fatty acid utilization [35]. Consistent with these findings, Chen et al. [37] showed in cultured second-trimester human cytotrophoblasts, that PFOA reduces cell viability and, at sub-cytotoxic concentrations, reprograms the expression of genes involved in lipid metabolism and innate immune signaling, including corticotropin-releasing hormone (CRH), interferon-induced protein with tetratricopeptide repeats 1 (IFIT1), and tumor necrosis factor superfamily member 10 (TNFSF10). Together, these findings indicate that PFASs interfere with tightly regulated trophoblast programs governing survival, differentiation, invasion, and metabolic support, processes fundamental to early placental establishment. Such sustained transcriptional reprogramming raises the possibility of longer-lasting regulatory effects mediated through epigenetic mechanisms.

### 3.3. Epigenetic Alterations and Sex-Specific Placental Susceptibility

Epigenetic mechanisms, including DNA methylation, histone modifications, and non-coding RNAs, regulate gene expression during embryogenesis and fetal development, periods particularly vulnerable to environmental perturbations [38,39]. The placenta appears particularly susceptible to PFAS-induced epigenetic disruption. Evidence from human studies indicates that prenatal PFAS exposure alters the placental epigenetic landscape, primarily through changes in DNA methylation [28]. Moreover, prenatal exposure to PFASs is associated with global histone methylation changes in 2-year-old children [40]. Several PFASs, including PFOA, PFOS, and PFHxS, have been associated with differential methylation of genes involved in placental development, cardiometabolic regulation, angiogenesis, and endocrine signaling [28,41]. While PFOA and PFOS often exert additive or cumulative effects, PFHxS displays distinct epigenetic signatures, highlighting compound-specific mechanisms of action [38]. The placenta appears particularly susceptible to PFAS-induced epigenetic disruption. Studies of gestational exposure reveal widespread alterations in placental DNA methylation, with PFOS and PFOA frequently acting synergistically on the placental methylome, whereas PFHxS, despite its use as a replacement compound, exhibits a divergent epigenetic profile [28], highlighting compound-specific mechanisms of action. Likewise, Xie et al. [41] reported PFDA was associated with increased solute carrier family 16 member 2 (SLC16A2) methylation, PFOA with decreased solute carrier organic anion transporter family member 1C1 (SLCO1C1) methylation, PFOS with increased TRH methylation, and perfluoroalkyl carboxylic acid (PFDoA) with decreased thyrotropin-releasing hormone (TRH) methylation. These findings emphasize that individual PFAS compounds, despite belonging to the same chemical class, exert distinct and compound-specific effects on gene-specific DNA methylation patterns. In addition, Toh and Sasaki [42] suggested that spatiotemporal genomic features shape megabase-scale DNA methylation patterns, including partially methylated domains (PMDs), in the human placenta and reveal clear differences in PMDs between human cytotrophoblasts and trophoblast stem cells. Recently, Sands et al. [43] using reduced representation bisulfite sequencing (RRBS), showed that PFOS exposure alters the epigenetic landscape by disrupting transcription factor binding sites related to tumorigenesis, inflammation, and stress responses. In the search for novel tools to reverse PFAS-induced damage, these authors showed that locus-specific CpG methylation editing in genes such as threonyl-TRNA synthetase 2, mitochondrial and mitogen-activated protein kinase 5 effectively reverses PFAS-induced toxicity and restores essential cellular functions [43]. Notably, placental epigenetic responses to PFASs are modulated by fetal sex. Prenatal exposure has been associated with reduced syncytiotrophoblast proportions in male placentas, whereas female placentas exhibit increased gestational epigenetic age acceleration, particularly in relation to PFOA and PFOS [44]. Sex-specificity effects were also noted by Xie et al. [41] in the associations between PFASs and DNA methylation. In addition, the authors identified compound-specific associations between individual PFASs and the differential regulation of thyroid hormone specific genes. Together, these findings indicate that placental susceptibility to PFASs is shaped by both chemical-specific properties and biological context. Importantly, epigenetic alterations in placental regulatory networks may translate into downstream metabolic and physiological consequences at the maternal–placental interface.

### 3.4. Maternal Placental Alterations and Redox Imbalance Associated with PFAS Exposure

The available evidence suggests that prenatal PFAS exposure is associated with alterations in maternal metabolic profiles, particularly during late pregnancy [45]. A meticulous metabolomic investigation of third-trimester serum has unveiled alterations in metabolites such as 2-hydroxybutyrate, along with components associated with branched-chain amino acids, energy metabolism and mitochondrial dysfunction. This compelling evidence points to a plausible disruption of fundamental metabolic pathways [45]. Complementing these findings, Cheng et al. [46] reported that PFOS exposure elevates levels of lysophosphatidylcholine and lysophosphatidylethanolamine in both maternal plasma and placental tissue. This suggests coordinated alterations in lipid metabolism across the maternal–placental interface. Taken together, these observations highlight the need for studies in larger and more diverse populations to clarify how PFAS-induced metabolic disturbances during pregnancy may contribute to fetal development vulnerability. At a mechanistic level, many of these metabolic alterations converge on pathways that regulate mitochondrial function and redox homeostasis.

Exposure to PFAS mixtures during early stages of pregnancy has been linked to significant changes in maternal serum metabolites, particularly those involved in oxidative stress, inflammation, and the metabolism of lipids and amino acids [39]. Altered metabolic pathways were found for glutathione, histidine, leukotriene, prostaglandin, linoleic acid, and vitamin (A, C, D, and E) metabolism, highlighting potential mechanisms through which exposure to PFASs may contribute to adverse maternal and placental outcomes [39]. Evidence suggests that PFASs disrupt cellular redox homeostasis by interfering with interconnected mitochondrial and metabolic pathways. This indicates that oxidative stress acts as an amplifying mechanism rather than a primary trigger [19,35,47]. In addition to directly increasing reactive oxygen species, several PFASs impair essential mitochondrial functions required for maintaining antioxidant capacity [35]. For example, research involving trophoblast-like HTR-8/SVneo cells and zebrafish embryos has shown that inhibits fatty-acid β-oxidation. This results in decreased tricarboxylic acid cycle activity and reduced availability of NADH and NADPH [47]. As NADPH is essential for regenerating glutathione, this metabolic constraint weakens cellular redox buffering and promotes oxidative stress [47]. Although Nuclear factor erythroid 2-related factor 2 (NRF2)-dependent antioxidant responses are activated as a compensatory mechanism in response to PFDA, they are insufficient to fully restore redox balance. This highlights mitochondrial dysfunction as a central driver of PFAS-induced oxidative stress in placental and embryonic tissues [47]. Distinct but complementary mechanisms have been described for other PFASs. Exposure to environmentally relevant concentrations of PFBS has been shown to alter placental bioenergetics by downregulating key regulators of lipid metabolism and mitochondrial fatty acid import. These include PPARG and components of the carnitine shuttle [35]. These changes lead to reduced ATP production despite increased mitochondrial DNA content and occur in the absence of overt oxidative stress or structural mitochondrial damage. This suggests, that PFBS mainly affects energy efficiency rather than causing conventional mitochondrial damage [35]. In contrast, PFOS has been shown to induce oxidative DNA damage in placental cells, as evidenced by elevated levels of 8-hydroxy-2′-deoxyguanosine [46]. While PFOS exposure also triggers NRF2-mediated antioxidant defenses, the persistence of DNA oxidative lesions indicates that these protective responses are inadequate to counteract sustained genotoxic stress [46]. Together, these findings illustrate that PFASs elicit compound-specific yet convergent mitochondrial and redox-disruptive effects. These effects undermine placental oxidative balance and metabolic resilience.

A summary of the effects caused by PFASs on trophoblasts and/or placenta are shown in Table 2.

## 4. Current Methodologies for the Analysis of Complex Chemical Mixtures

### 4.1. Analytical Challenges and Advances in PFAS Biomonitoring

Despite the consistent detection of PFASs in biological matrices at low ng/mL concentrations, typically ~1–3 ng/mL for PFOA and ~2–5 ng/mL for PFOS in population-based studies of pregnant people, biomonitoring remains analytically challenging due to low analyte abundance and substantial matrix interferences [17]. Targeted liquid chromatography–tandem mass spectrometry (LC–MS/MS) currently represents the gold standard for PFAS quantification; however, methodological limitations persist, particularly for short-chain and highly polar compounds [17]. Beyond mass spectrometry-based approaches, emerging biosensing, such as protein-based electrochemical sensors capable of point-of-need detection of PFOA at sub-ng/L levels in water and whole blood, offers a complement to conventional LC–MS/MS [48]. Recent methodological advances, including optimized extraction solvents and salt compositions, have improved PFAS recovery and enabled efficient extraction and clean-up across diverse biological matrices [49]. Nevertheless, growing evidence indicates that targeted approaches alone may be insufficient under unknown or complex contamination scenarios. Analytical interferences affecting the quantification of compounds such as perfluorobutanoic acid (PFBA) and perfluoropentanoic acid (PFPeA) have been reported in both biological and environmental samples, raising concerns regarding exposure misclassification [50,51]. Consequently, high-resolution mass spectrometry with non-target and suspect screening has emerged as a complementary strategy, enabling broader PFAS characterization and exposure assessment, particularly for emerging PFASs and structurally similar analogues [52]. Buckenmaier et al. [53] demonstrated that a feed-injection two-dimensional LC–MS approach enhances PFAS quantification in complex matrices by increasing analytical sensitivity, reducing matrix effects, and allowing more accurate detection of short-chain compounds such as PFBA. Additionally, differential mobility spectrometry improves PFAS resolution and discrimination among isomeric and structurally related compounds, albeit with slightly higher limits of quantification compared with conventional LC–MS/MS [54]. Recent advances in online SPE–UHPLC–HRMS (solid-phase extraction/ultra-high-performance liquid chromatography/high-resolution mass spectrometry) have enabled sensitive, high-throughput, and simultaneous quantification of legacy and emerging PFASs in human serum at low ng/L levels, supporting robust exposure assessment and associations between PFAS exposure and adverse pregnancy outcomes [8].

### 4.2. Use of New Approach Methodologies (NAMs)

As we are constantly exposed to many chemicals, the field of toxicology is shifting towards analyzing complex chemical mixtures that are more relevant to real-life exposure situations. Depending on whether substances can interact with each other, different approaches to mixture risk assessment can be followed [55]. In this regard, the use of new approach methodologies (NAMs) is currently encouraged. For instance, the Bayesian Kernel Machine Regression (BKMR) is employed to analyze nonlinear dose-response curves and the interaction between different chemicals, while Weighted Quantile Sum (WQS) regression is utilized to evaluate the relative contribution of each chemical to the total toxic effect [31,56,57]. A 2020 study using these approaches found an association between exposure to a PFAS mixture during early pregnancy and impaired maternal and neonatal thyroid function. The study estimated the individual and joint effects of PFASs and found that PFASs may have a combined effect on thyroid function [31]. Another study used quantile g-computation and BMKR to study the joint effects of seven PFASs in relation to markers of oxidative stress during pregnancy [58]. Quantile g-computation estimated the overall change in an oxidative stress biomarker, analyzing the effect of components of the mixture on the outcome, reflected with relative weights. Then, the BMKR identified linear and nonlinear associations and interactions between the exposures [58]. This allowed for the characterization of partial and cumulative dose-response relationships. Other methodologies include concentration addition models, which assume that mixture components act additively. These models are used to predict the combined potency of a mixture by summing the fractional potency of its constituents [59]. This model has been used in pregnancy-related experiments involving Sprague Dawley rats exposed to PFOA and PFOS. It was found that combined exposure had cumulative effects on both mothers and their offspring [60]. Physiologically Based Kinetic (PBK) models simulate the absorption, distribution, metabolism, and excretion of chemicals using information about the organism’s physiology and the chemical properties, thereby allowing for the estimation of ‘dose-at-target’ [61]. Some studies have used PBK models to investigate the uptake and distribution of PFASs in humans [62,63,64]. PBK models have been used to study the effects of PFASs in animal models. For example, Golosovskaia et al.’s study [61] analyzed the uptake of seven emerging PFASs, as well as their potential for maternal transfer in zebrafish. The study found that all the PFASs studied (PFNA, PFOA, PFBS, PFHxS, perfluorooctane sulfonamide, and 6:2 perfluorooctane sulfonate) were transferred to the eggs via the mother. Paré et al. [65] recently used a mouse-specific physiologically based pharmacokinetic (PBPK) model to study the effects of PFOA. This model incorporated a placental structure to model growth and volume changes in the placenta and fetal litter during pregnancy, enabling the prediction of PFOA kinetics in pups [65]. These models provide an important tool for understanding kinetics under complex mixture scenarios. Other NAMs involve hybrid methods that combine machine learning (AI-HNN) and a pathophysiology method (CPTM) to predict toxicity in a dose-dependent manner, as well as specific organ risks [66]. To our knowledge, no studies using this hybrid method have yet been conducted in relation to analyzing the effects of PFASs on the placenta. As part of NAMs, in vitro assays such as cell culture (either 2D or 3D), in silico modelling and omics technologies are also encouraged [67]. Some of these NAMs have been used to analyze the effects of different PFASs on trophoblasts or placentas, as already shown in Section 3.4 and summarized in Table 2. Experiments using trophoblast spheroid models are particularly well-suited to studying the effect of complex mixtures of PFASs on placental function [33]. In another recent study, Moyer et al. [68] used a four-cell human fetal–maternal interface organ-on-chip (FMi-OOC) model to mimic in utero tissue topology. They added PFOA to the maternal (decidua) chamber to study cell viability and cytokine production in the different FMi-OOC chambers (decidua, chorion trophoblast, amnion mesenchymal and amnion epithelial cells). Their study suggests that exposure to PFOA leads to the occurrence of a pro-inflammatory state in the fetal membranes, which could be a trigger for preterm birth [68].

**Figure 1:**
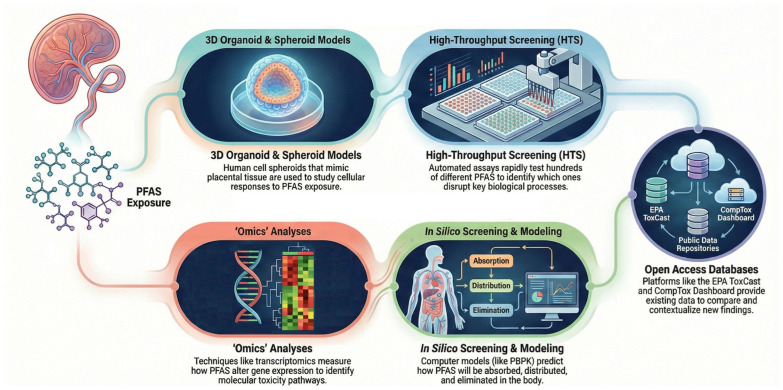
Schematic representation of the possible use of NAMs to study the global effect of mixtures of PFASs on the placenta. NAMs: New approach methodologies.

Although different NAMs have already been used to study the effects of various PFASs, further investigation is needed to fully understand the effects on the placenta and developing fetuses. Moreover, the complexity of the fetal-maternal interface, coupled with the complexity of the various PFAS mixtures, poses a challenge in developing a NAM capable of covering the potential scenarios and outcomes. In that regard, the integration of data from open-access resources, such as the EPA’s ToxCast or the CompTox Chemicals Dashboard together with the use of NAMs, is currently encouraged [69]. A comprehensive schematic representation of the different kinds of NAMs that could be used to explore the effects of mixtures of NAMs is depicted in Figure 1.

## 5. Discussion

This article reviews the potential impact of PFAS exposure, emphasizing the importance of regulating the level of exposure to chemicals for ensuring the healthy development of a population. Although exposure occurs involuntarily throughout the lifespan, there are critical periods of vulnerability, with pregnancy being one of the most sensitive. In this context, studying the effects of chemicals on the placenta is important, as although the placenta partially shields the fetus from toxic substances, the effects of toxic chemicals on the placenta may be crucial to fetal well-being.

Per- and polyfluoroalkyl substances (PFASs) are a large group of fluorinated chemicals widely used in consumer products and by industry, due to their unique thermal and chemical stability, which contributes to their environmental persistence. Exposure to long-chain PFASs such as PFOS and PFOA have been linked to developmental [70], immune [71] and reproductive toxicity [72]. Even though they are now banned or strictly regulated, they are considered persistent organic pollutants under the Stockholm Convention [73] and their presence is still of concern. In addition, a vast number of PFASs are still being produced and used, making the PFAS landscape immense (over 15,000 substances [74]). Regarding the effects of PFASs on the placenta, numerous studies have focused on the effects of some of the most ubiquitous PFASs.

Epidemiological studies have demonstrated an association between PFASs and adverse pregnancy outcomes, such as low birth weight [11], preeclampsia [21] and gestational diabetes [75]. While some authors have found these associations, other authors do not find a clear association between certain PFASs and adverse pregnancy outcomes [76,77]. For example, some authors have associated higher concentrations of PFASs during pregnancy with a lower risk of gestational hypertension [78], whereas other authors have found no evidence of an increased risk of gestational hypertension, preeclampsia, or gestational diabetes [77]. Most of these discrepancies in epidemiological associations may arise from the difficulties in analyzing different PFASs that may be present in the samples but not considered or not detectable due to analytical challenges.

The different associations of PFASs with adverse pregnancy conditions are closely related to their effects on the placenta. These effects are strongly influenced by their capacity to cross the placental barrier and the placenta’s capacity to bioaccumulate PFASs. This exposes the fetus to PFAS concentrations comparable to those in the mother’s blood [79,80]. The efficiency of placental transfer varies among different PFAS congeners. For example, the length of the chain appears to influence transfer efficiency; shorter-chain PFASs such as PFHxS and PFOA generally exhibit higher transfer efficiencies than longer-chain sulfonates such as PFOS [14,15]. Studies have found maternal-to-cord serum ratios of around 1:1 for PFOA and 2:1 for PFOS, suggesting that PFOA is more easily transferred [5]. Isomerism also appears to play a role, with linear PFAS structures crossing the placental barrier more efficiently than branched isomers [81,82]. Regarding emerging PFASs, ether-linked PFASs such as GenX and 6:2 Cl-PFESA are efficiently transported too, with some of them showing higher transfer rates than legacy compounds [15,26]. Therefore, even though some PFASs are now strictly regulated, the fact that they persist in the environment and that emerging PFASs can also cross the placental barrier poses a significant toxicological risk. Furthermore, exposure to a mixture of different PFASs simultaneously poses an additional analytical challenge that can be addressed using NAMs.

Different types of NAMs have been used to analyze the effects of complex PFAS mixtures on placental function. Some of these models use cell lines and enable the simultaneous evaluation of multiple PFASs. While they are valuable for analyzing complex mixtures, there are limitations that need to be overcome. For instance, concentrations used in vitro may not accurately reflect potential in vivo effects, making in vitro-to-in vivo extrapolation models necessary for risk assessment [83]. Furthermore, cell lines are less complex than in vivo mammalian test systems [84]. This may result in the true risk of compounds, for which toxicity is highly dependent on biotransformation capacity, being underestimated [83]. Another point to consider is the long lifespan of many PFASs, which complicates risk assessment. Their persistence in the environment, widespread dispersion and accumulation in living organisms lead to bioaccumulation and biomagnification, introducing an element of uncertainty to risk estimates [85]. Additionally, the different toxicokinetic properties of PFASs must be considered to understand issues such as bioaccumulation and species/sex differences in response [86]. The review of Deepika et al. [87] addresses these issues, pointing out that although PBPK models are suitable for some PFASs, they still need to be further optimized and validated. Novel approaches must take into account tissue-specific modes of action, with a focus on molecular interactions involving enzymes, storage and transport proteins, in order to define their kinetics [87]. The integration of data from open-access resources with NAMs that take these aspects into consideration, could help to close the knowledge gap regarding the effects of different combinations of PFASs on the placenta and during pregnancy.

Overall, this review emphasizes the importance of the placenta as a target for PFAS toxicity, highlighting the molecular and functional disruptions that may hinder fetal development and lead to adverse pregnancy outcomes. Integrating new approach methodologies, such as placental organoids, omics-based analyses, and high-throughput screening platforms, provides more human-relevant and mechanistically informative tools for assessing the risk of PFASs. Together, these advances strengthen the evaluation of maternal–fetal exposure, improve the identification of emerging PFASs of concern and support the development of more protective regulatory strategies.

## Figures and Tables

**Table 1:** Summary of different PFASs found in biological samples indicating prenatal exposure to a variety of different PFASs.

PFAS Category	Compound Abbreviations	Maternal Serum/Plasma	Fetal Serum	Umbilical Cord Blood	Placenta	References
Legacy PFASs	PFOS, PFOA, PFHxS, PFNA	✔	✔	✔	✔	[5,11,15,16,17,18,21,22,23,24,30,31]
Legacy PFASs (placental-focused studies)	PFOS, PFOA, PFHxS	—	—	—	✔	[11,14,28,29]
Emerging PFASs (short-chain)	PFBS, PFHxA, PFBA, PFHpA	✔	—	✔	✔	[13,17,27]
Emerging PFASs (ether based/chlorinated)	HFPO-DA (GenX), Cl-PFESA (F-53B)	✔	—	✔	✔	[6,17,27]
Emerging PFASs (review evidence)	Multiple emerging PFAS	✔	✔	✔	✔	[12,17]

Abbreviations: Cl-PFESA (F-53B): chlorinated polyfluoroalkyl ether sulfonic acid; HFPO-DA (GenX): hexafluoropropylene oxide dimer acid; PFBA: perfluorobutanoic acid; PFBS: perfluorobutane sulfonic acid; PFHpA: Perfluoroheptanoic acid; PFHxA: perfluorohexanoic acid; PFHxS: perfluorohexane sulfonic acid; PFNA: perfluorononanoic acid; PFOA: perfluorooctanoic acid; PFOS: perfluorooctane sulfonate.

**Table 2:** Summary of PFAS effects on placental development.

PFAS	Experimental Model	Primary Effects on Trophoblasts or Placentas	Key Molecular Pathways/Mechanisms Affected	Reference
PFOA, PFOS, GenX	JEG-3 monolayers	Diminished viability, proliferation, mitochondrial membrane potential; diminished migration	Oxidative stress genes (GPX1, SOD1), xenobiotic transport (ABCG2)	[32]
PFAS mixture	JEG-3 spheroids	Structural damage at high dose; high invasion at low dose	Apoptosis, invasion related signaling	[33]
PFAS mixture	HTR-8/SVNeo spheroids	Consistent diminished invasion at low-medium doses	Apoptotic balance (CASP3), immune modulation (MIF)	[33]
PFAS mixture	JEG-3/HTR-8	Diminished β-hCG secretion; diminished CBG7 expression	Endocrine signaling; CREB-dependent transcription	[33]
PFOA, PFOS	Human trophoblast organoids	Altered lineage specification, diminished hormone secretion; diminished invasion	CREB-signaling; differentiation programs	[34]
PFBS	Placental cells (integrated omics)	Impaired lipid metabolism; diminished mitochondrial fatty acid utilization	PPARG; carnitine shuttle (CPT1A, SLC25A20)	[35]
PFOA	Primary human cytotrophoblasts (2nd trimester)	Diminished viability; transcriptomic reprogramming at sub-cytotoxic doses	Lipid metabolism; innate immune genes (CRH, IFIT1, TNFSF10)	[37]
PFOA, PFOS, PFHxS	Human placenta (epigenome-wide)	Differential DNA methylation; sex-specific effects	Developmental, cardiometabolic and endocrine pathways	[28]
PFDA	HTR-8/SVNeo; zebrafish embryos	Diminished β-oxidation; augmented oxidative stress	Mitochondrial metabolism; NRF2 response	[47]
PFOS	Placental cells; human samples	Oxidative damage	Redox imbalance; genotoxic stress	[46]

Abbreviations: ABCG2: ATP-binding cassette subfamily G member 2; CASP3: caspase-3; CPT1A: carnitine palmitoyltransferase 1A; CREB: cAMP response element-binding protein; CRH: corticotropin-releasing hormone; GenX: hexafluoropropylene oxide dimer acid; GPX1: glutathione peroxidase 1; IFIT1: tetratricopeptide repeats 1; MIF: macrophage migration inhibitory factor; NRF2: nuclear factor erythroid 2-related Factor 2; PFAS: poly- and perfluoroalkyl substances; PFBS: perfluorobutane sulfonic acid; PFDA: perfluorodecanoic acid; PFHxS: perfluorohexane sulfonic acid; PFOA: perfluorooctanoic acid; PFOS: perfluorooctane sulfonate; PPARG: peroxisome-proliferator-activated receptor gamma; SLC25A20: solute carrier family 25 member 20; SOD1: superoxide dismutase 1; TNFSF10: tumor necrosis factor superfamily member 10.

## Data Availability

No new data were created or analyzed in this study. Data sharing is not applicable to this article.
